# Characterization and genomic analysis of a *Herelleviridae* bacteriophage UHP46 infecting mastitis-causing *Staphylococcus aureus*

**DOI:** 10.3389/fmicb.2025.1496919

**Published:** 2025-02-12

**Authors:** Sara Najeeb, Imran Khan, Javed Muhammad, Muhammad Jahangir, Iqbal Ahmad Alvi, Anza Abbas, Aman Ullah, Arif Ullah, Wajiha Sajjad, Hashir Khan, Amjad Khan

**Affiliations:** ^1^Department of Food Science and Technology, University of Haripur, Haripur, Pakistan; ^2^Department of Microbiology, University of Haripur, Haripur, Pakistan; ^3^Department of Microbiology, Hazara University, Mansehra, Pakistan; ^4^Department of Public Health and Nutrition, University of Haripur, Haripur, Pakistan; ^5^Department of Veterinary sciences, University of Kentucky, Lexington, KY, United States

**Keywords:** mastitis, *Staphylococcus aureus*, antibiotic resistance, bacteriophages, phage therapy

## Abstract

**Background:**

Mastitis is a widespread disease on a global scale, significantly impacting the dairy industry. Mastitis in dairy cattle is caused by over 150 different bacteria, with *Staphylococcus aureus* (*S. aureus*) playing a significant role in financial losses, problems with animal welfare, and challenges with food safety. Phage treatment is thus being investigated as an effective replacement for reducing contaminants and illnesses caused by bacteria. In this study, we identified a phage UHP46, that effectively targets mastitis-causing *S. aureus*.

**Methods:**

*S. aureus* S46 was used to screen for the wastewater lytic phages. The isolated lytic phage UHP46, which formed clear plaques and spots, was further characterized.

**Results:**

Phage UHP46, belonging to the *Herelleviridae* family, forms clear, circular plaques in bacterial lawn. UHP46 showed stability under various range of temperature and pH levels, with maximum activity observed at pH 7 and temperature 37°C. Genomic analysis revealed that phage UHP46 is a dsDNA virus with an approximate genome size of 139,731 bp, and it encodes 72 proteins with known functions and 136 hypothetical proteins. One-step growth curve analysis indicated latent period of approximately 20 mins and burst size of about 27 progeny/cell. In organic stability test, UHP46 showed stability in DMSO and acetone. Furthermore, it effectively inhibited *S. aureus* growth for up to 16 h, suggesting its suitability for therapeutic applications against *S. aureus* infections.

**Conclusion:**

These findings suggest that phage UHP46 could serve as a promising alternative to antibiotics for managing *S. aureus*- induced mastitis, contributing to dairy production and improved animal health.

## 1 Introduction

Bovine mastitis, recognized by mammary gland’s inflammation, results from microbial infection or physical injury (Zhao and Lacasse, 2008). It is responsible for poor quality and reduced yield of milk, which accounts for substantial economic loss in dairy industries (Moschovas et al., 2023). Bovine mastitis is predicted to have an annual cost of failure of approximately 147 dollars linked to each cow, globally, with culling and milk yield associated losses making up 11–18 percent of the profit margin coming from each cow (Hogeveen et al., 2019). Decreased milk production due to mammary tissue damage results in 70% of the total losses (Zhao and Lacasse, 2008).

Mastitis is caused by a variety of pathogens, with *staphylococci*, *enterobacteria*, and *streptococci* accounting for the majority of infections (Haxhiaj et al., 2022). Mastitis is generally divided into two forms, clinical and subclinical infections (Cao et al., 2023), and the pathology might be environmental or contagious, based on the method of transmission and primary reservoir (Gomes et al., 2016). The main environmental pathogens causing mastitis include *Klebsiella* spp., *Escherichia coli*, and *Streptococcus uberis*. On the other hand, *Streptococcus agalactiae* and *S. aureus* are the examples of contagious microorganisms, and their main source is the infected mammary gland of cows (Gomes and Henriques, 2016). In dairy animals, *S. aureus* is most commonly associated with mastitis, which is hard to treat and also presents high risk of reoccurrence. Researchers have recognized *S. aureus* as the leading cause of mastitis causing pathogen due to its various characteristics such as higher antibiotic resistance, significant virulence factors, and the capacity to induce long-term infections (Hoque et al., 2018). *S. aureus* uses diverse virulence factors such as different toxins (exfoliative toxins Eta, Etb, toxic shock syndrome toxin-1 TSST-1, and enterotoxins SEA to SEQ), cell surface structures (intercellular adhesion genes A and D, and clumping factor A clfA), and immune evasion molecules (coagulase, protein A, leucocidins, and haemolysins) (Monistero et al., 2018).

Frequent mastitis infections in dairy animals lead to the increased use of antibiotics. The unregulated and unchecked consumption of antibiotics results in the development and spread of resistant bacteria (White and McDermott, 2001). Moreover, bacteria residing intracellularly within the mammary gland are challenging to treat due to their limited contact with antibiotics. In case of *S. aureus*, the current rate of cure with licensed antibiotics (such as pirlimycin) is about 10–30% (Gomes and Henriques, 2016). In addition, ability of pathogenic bacteria to grow as biofilms presents a key factor attributed to recurrence of mastitis infections and antibiotic resistance (Olson et al., 2002). Therefore, continuous rise in antibiotic-resistant bacteria urges scientists to develop alternative therapeutic strategies, and phage therapy has shown high efficacy in eradicating resistant pathogens (Li and Zhang, 2014). Bacteriophages, commonly called as phages, are the viruses which target bacterial cells by delivering their genomic content inside the host (Jamal et al., 2019). Phages are classified as lysogenic or lytic on the basis of their life cycle (Inal, 2003). They are found in almost every environment, for instance, ocean water is believed to have approximately 10^7^ phage particles per milliliter (Wommack and Colwell, 2000), and total estimated phage count in biosphere is about 10^31^ (Mushegian, 2020). Phages offer several advantages over antibiotics based on their high host specificity, which allows them to protect the normal microflora (Kutateladze and Adamia, 2010).

The potential of phages has been investigated against *S. aureus* as biocontrol agents in food products (O’Flaherty et al., 2005; Wen et al., 2023), and as therapeutic agents in controlling mastitis (Geng et al., 2020). A number of investigations have proved that phages possess strong bactericidal potential against MDR strains of *S. aureus* (Varela-Ortiz et al., 2018), and phage testing in animal models infected with *S. aureus* has also shown promising result (Breyne et al., 2017). Continuous growth of phages in natural environments allow them to exhibit significant genetic diversity and highly dynamic nature, which ultimately allows researchers to explore different phages for MDR bacteria (Hatfull et al., 2022). Accordingly, the main aim of this study was to investigate the efficacy of lytic phages against mastitis-causing *S. aureus*, and determine the effect of different environmental conditions on the activity of phage. The genomic analysis of phage was also performed to better understand its phylogenetic association and structural components.

## 2 Materials and methods

### 2.1 *Staphylococcus aureus* isolation and characterization

Following the guidelines set out by the National Mastitis Council (NMC 2001), milk samples were aseptically collected from dairy farms in the regions of Khyber Pakhtunkhwa, Pakistan (NMC 2001). A milk sample (1 mL) was transferred to a California Mastitis Test (CMT) paddle, and the same amount of CMT reagent (Immucell, United States) was added. The mixture was gently swirled for 20–30 s, after which results were recorded according to the protocol. The CMT-positive milk samples were further processed for microbiological analysis. To isolate *S. aureus* from milk, samples were enriched by incubating 1 mL of milk with 5 mL of Brain Heart Infusion broth and incubated the mixture for 24 h at 37°C (Hegde et al., 2013). An aliquot of each milk sample (20–100 μL) was spread on Mannitol Salt agar to isolate Gram-positive bacteria. Gram staining, coagulase, catalase, Voges-Proskauer (VP), urease, indole, and methyl Red tests were performed to confirm the presence of *S. aureus* (Talaiekhozani et al., 2015). Using the phenol-chloroform isoamyl alcohol method, genomic DNA was extracted from all biochemically verified *S. aureus* strains. Additionally, 16S rDNA sequence analysis was carried out to identify microbes (Saruta et al., 1997). In accordance with CLSI 2019 standards, the Kirby-Bauer disk diffusion assay was used to evaluate the antibiotic susceptibility profile of the *S. aureus* isolates (Humphries et al., 2019).

### 2.2 Isolation of phage

Water samples were collected from various sources, including wastewater and animal farms, to obtain *S. aureus* specific phage. The water samples were centrifuged at 21,380 × g for 15 min to separate the phage from other particles. Then the supernatant was filtered through 0.45 μm syringe filter, and the filtrates were stored at 4°C for further processing (Emencheta et al., 2022).

### 2.3 Phage enrichment and isolation

A bovine *S. aureus* strain, S46, isolated from raw milk was cultured on Nutrient agar and incubated at 37°C for 24 h. After the incubation, colonies were transferred to Nutrient broth and incubated further for 24 h. Then, 20 mL of bacterial culture was mixed with 25 mL of wastewater supernatant. An additional 20 mL of Nutrient broth was added to the mixture, which was then incubated at 37°C for 24 h at 200 rpm in a shaking incubator (Heidolph Unimax 1010).

After incubation, the mixture was centrifuged at 7,024 × g for 15 min at 4°C. The supernatant was filtered through 0.22 μm syringe filter. For the initial confirmation of phage in the sample, spot assay was done. Briefly, the bacterial lawn was prepared by mixing 3 to 5 mL of 0.7% semi-solid agar (containing peptone 5 g/L, beef extract 3 g/L, sodium chloride 5 g/L, agar 7 g/L) with 100 μL of an early exponential phase bacterial culture. This mixture was then poured onto pre-prepared Nutrient agar plates, spread evenly to cover the surface, and allowed to dry for 3–5 min. Using a pipette, 10 μL aliquots of phage samples were spotted on the agar overlay surface. The plates were incubated at 37°C for up to 24 h. Clear zone on the bacterial lawn was presumptive of specific phage presence. Plaque assay was carried out for purification and subsequent quantification of phage (Alvi et al., 2020).

### 2.4 Purification of phage

Phage was purified by picking a single clear plaque with the help of a sterile tip followed by re-suspension in SM buffer. The phage was re-propagated by mixing the suspension with the log phase host bacteria followed by centrifugation and filtration. Through double layer agar method, plaque assay was performed 10–12 times, and the phage titer was expressed as plaque-forming units per milliliter (PFU/mL). The isolated phage was stored in Nutrient broth at 4°C for short-term storage, and − 80°C for long-term storage with 50% glycerol added (Alvi et al., 2020).

### 2.5 Morphological analysis of UHP46

Morphological features of phage UHP46 were determined using transmission electron microscopy (TEM). Briefly, the purified phage lysate (15 μL) was loaded on to carbon coated copper grids, allowed to dry, and treated with phosphotungstic acid (PTA). After removing the excess liquid, the stained copper grid was observed under FEI T20 transmission electron microscope (FEI, Hillsboro, OR).

### 2.6 Determination of the potential of phage to prevent bacterial proliferation

*In vitro* reduction of bacterial growth by phage was determined using a bacterial growth reduction assay. UHP46 (3 × 10^7^ PFU/mL) was added to S46 strain cultured in Nutrient broth at multiplicity of infection (MOI) of 1, and the mixture was incubated with shaking at 37°C. The bacterial growth reduction ability of UHP46 was monitored by measuring optical density at 600 nm (OD_600_) every 2 h, with the growth of S46 control, compared over a 16-h period (Khawaja et al., 2016). The data was analyzed through non-parametric Mann–Whitney test using GraphPad prism software, and the *p*-values were calculated using a *T*-test.

### 2.7 Determination of pH and thermal stability

The pH stability of UHP46 was evaluated by adjusting SM buffer to pH levels of 2, 4, 6, 7, 8, 10, and 12 using either 1 M HCl or 1 M NaOH, with a known titer of 4.0 × 10^7^ PFU /mL. The phage was then suspended in 1 mL of adjusted SM buffer and incubated for 1 h. The pH was then neutralized by preparing serial dilutions in SM buffer (pH 7) and the phages were enumerated by double layer agar method (Hammerl et al., 2014). For thermal stability assessment, 1 mL aliquots of UHP46 having viral titer of 9 × 10^7^ PFU/mL were exposed independently to a range of temperatures (20, 25, 30, 37, 45, 55, 65, 75, and 85°C) for 1 h. Phage titer was calculated through plaque assay afterward to evaluate temperature effects on stability (Asif et al., 2020). The data for pH and thermal stability of UHP46 was analyzed using GraphPad prism software, and the p-values were calculated using a one-way ANOVA test.

### 2.8 One-step growth curve assay

One-step growth curve assay for UHP46 was performed as previously described (Vukotic et al., 2020). The experiment was performed at an MOI of 0.01 (3 × 10^9^ CFU/mL and 3 × 10^7^ PFU/mL). The bacteria and phage were added in equal volumes (500 μL each) and incubated at 37°C for 5 min followed by centrifugation at 13,000 × g for 1 min. The supernatant was discarded and the pellet was suspended in 100 mL Nutrient broth. During the subsequent 30-min incubation (starting from the point of dilution), 1 mL sample was collected at every 5 min interval and the phage titer was determined by double layer agar overlay method. The experiment was repeated three times, and burst size was subsequently calculated.

### 2.9 Determination of organic solvent stability

The stability of UHP46 in various organic solvents was determined by the method previously described by Fikadu et al. (2024) with some modifications. After mixing an equal volume of phage UHP46 with ethanol, methanol, DMSO, acetone and xylene, the mixture was incubated for 1 h at 37°C. As a control, SM buffer was mixed with phage in equal volumes. Once the phage and solvent combination had been incubated, the phage titer was determined using the double agar-layer technique (Fikadu et al., 2024).

### 2.10 Phage host range determination

The host range of UHP46 against several pathogenic strains of *S. aureus* was determined by modifying the approach by Alvi et al. (2020). The activity of UHP46 was checked using the double agar overlay method, and the plates were examined to determine if any inhibition zones had formed. The inhibition zones were categorized as clear, turbid, or absent. The activity of the phage was tested against 29 *S. aureus* strains isolated from bovine mastitis milk.

### 2.11 Evaluation of UHP46 phage stability in heat-treated milk

The stability of phage UHP46 in heat-treated milk was evaluated as previously described, with some modifications (Ávila et al., 2023). Briefly, raw milk was sterilized by heating the milk to 110°C for 20 min and subsequently exposing it to UV light at 245 nm for 15 min to remove contaminants. An initial check for bacterial load in milk using serial dilution and culturing on MSA media was performed. Sterile test tubes containing 1 mL milk with a final phage titer of 2 × 10^9^ PFU/mL were incubated at 4°C, 25°C, and 37°C. After 4 h and 24 h, phage aliquots were taken for double-layer agar assay to determine the phage titer.

### 2.12 Whole-genome sequencing analysis

The phenol-chloroform-isoamyl alcohol (PCI) method was used to extract phage DNA (Sambrook and Russell, 2006). For this purpose, DNase I (1 U) and RNase A (100 μg) were mixed with a 1 mL aliquot of filtered phage lysate (4.6 × 10^12^ PFU/mL), and the mixture was incubated for 4 h at 37°C. The mixture was placed in a water bath at 75°C for 10 min in order to denature the enzyme (DNase and RNase). Next, 50 μL of 10% SDS, 2.5 μL of proteinase K (20 mg/mL), and 40 μL of 0.5 M EDTA were added to the mixture. The sample was then incubated for 1 h at 55°C, with 20-min intervals of gentle mixing. After this, two microcentrifuge tubes containing equal amounts of lysate and PCI were centrifuged for 10 min at 12,000 × g. The top layer was carefully removed and transferred to a new microcentrifuge tube after centrifugation. Next, 50 μL of 3 M sodium acetate and 1 mL of ice-chilled 95% ethanol were added to each tube. After placing on ice for 5 min, the tubes were centrifuged again for 10 min at 12,000 × g. Subsequently, the pellet was rinsed with 500 μL of 70% ethanol and centrifuged at 12,000 × g for 5 min. The tubes were inverted and allowed to dry on blotting paper after the supernatant was disposed. After that, the DNA pellet was dissolved for 15–20 min in 50 μL of nuclease-free water.

Whole-genome sequencing was performed commercially by Macrogen Korea on an Illumina sequencing platform. SPAdes (4.0.0) was employed in assembling sequencing reads obtained after sequencing of whole genome. RAST server was used to annotate the assembled genome (Aziz et al., 2008). The predicted open reading frames (ORFs) were further analyzed through Interproscan,^1^ and BLASTp.^2^ Proteins containing transmembrane helices and signal peptides were identified using TMHMM,^3^ and SignalIP-5.0.^4^ Genes encoding for tRNAs were scanned by using tRNAscan-SE v. 2.0.^5^ Isoelectric point and molecular weight of the UHP46 proteins was calculated through the Expassy ProtParam tool.^6^ The PhageTerm program, which is part of Galaxy CPT Public^7^ was used to identify genomic termini and predict the head packaging mechanism employed by phages. BACPHLIP, integrated in Galaxy CPT Public (see Footnote 7) was used to assess the virulence score of UHP46. UHP46 genome was uploaded to the NCBI GenBank database with assigned accession number PP995776. VICTOR database^8^ and MEGA11^9^ were used for phylogenetic analysis of UHP46 compared with other related phages. Furthermore, PhageAI^10^ and Phagenomics^11^ were used to identify genomic features of UHP46. Antibiotic resistance, and virulence associated genes in UHP46 genome were screened by using ResFinder 4.6.0,^12^ and VirulenceFinder 2.0,^13^ respectively.

## 3 Results

### 3.1 Isolation of phage and morphology determination

**Figure 1:**
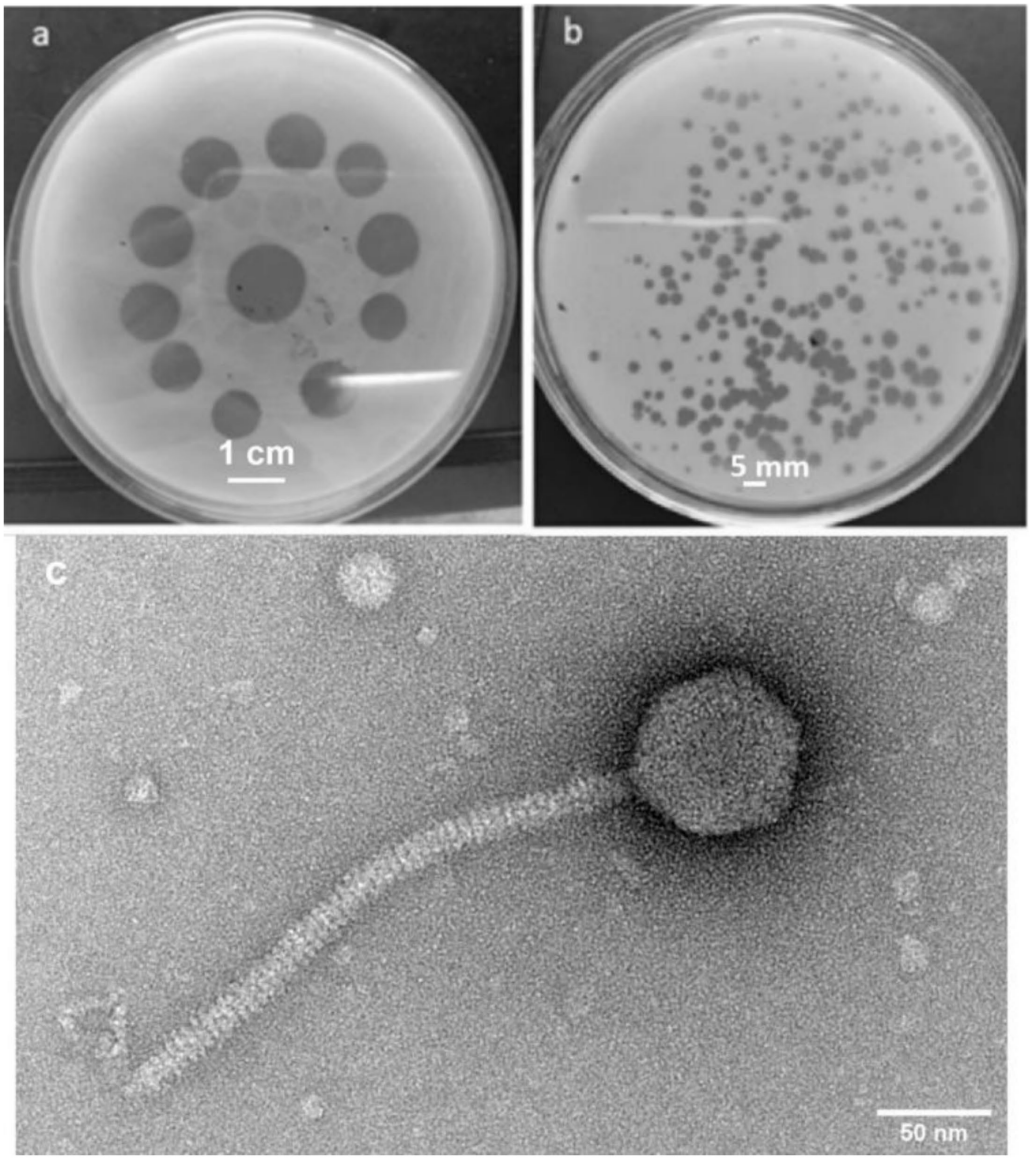
Phage UHP46 plaques on a *S. aureus* lawn. **(a)** The spot test showed a distinct lysis region. **(b)** After the purified phages were plated on double-layer agar, clear plaques were observed. **(c)** Transmission electron microscopic image of UHP46 shows long tail, with an icosahedral head.

Naturally occurring strains of *S. aureus* were identified to determine the most effective phage for the controlling or preventing bovine mastitis. *S. aureus* isolates were obtained from raw milk samples collected from dairy farms with cases of clinical mastitis and antibiotic susceptibility profile of the *S. aureus* isolates is presented in Table 1. A virulent phage UHP46 specific to S. aureus S46 was isolated from wastewater, showing clear spots on the bacterial lawn (Figure 1A). UHP46 produced transparent, circular plaques with a diameter of 1–2 mm, as seen in Figure 1B. The single plaque was propagated for further physiological and genetic analysis, after purification based on their morphology. TEM analysis indicated that UHP46 has a long, tail 170 nm in length, and icosahedral head with approximately 52 nm in diameter (Figure 1C). Based on these morphological features, UHP46 can be classified as a member of *Siphoviridae* family of the class *Caudovirales* phage.

**Table 1:** Sensitivity of bacterial isolates against antibiotics.

*S. aureus*	Antibiotics									
Sample No.	Fox 30	NOR 10	CN 10	P 10	DA 2	E 15	LZD 30	CIP 5	TE 30	Total resistance
11	R	I	R	R	R	R	R	I	I	6
16B	R	I	R	R	R	R	R	I	R	7
20A	R	S	R	R	R	R	R	I	R	7
20B	R	S	R	R	R	R	R	I	I	6
25	R	S	R	R	R	R	R	I	R	7
27	R	S	R	R	R	R	R	I	R	7
28	R	S	R	R	R	R	R	R	S	7
30	R	S	R	R	R	R	R	I	R	7
39	R	S	–	R	R	R	R	I	R	6
43	R	S	R	R	R	R	R	S	R	6
44	R	S	S	R	R	R	R	I	R	6
46A	R	S	I	R	R	R	R	S	R	6
S46	R	S	S	R	R	R	R	I	R	6
46C	R	S	R	R	R	R	R	S	R	7
47B	R	S	–	R	I	R	R	S	S	4
47C	R	S	–	R	I	R	R	S	S	4
48A	R	S	–	R	I	R	R	S	S	4
48B	R	S	–	R	I	R	R	I	S	4
48C	S	S	–	R	I	S	S	S	S	1
49	S	S	–	R	I	I	S	S	S	1
50A	R	S	–	–	R	R	R	S	S	4
50C	S	S	–	R	I	R	S	S	–	2
54B	S	S	–	R	I	I	S	S	–	1
60B	R	S	–	R	R	R	R	S	S	5
61B	R	S	–	R	R	R	R	S	I	5
94	S	S	S	S	S	S	S	S	S	0
90	S	S	S	S	S	S	S	S	S	0
96B	S	S	S	S	S	S	S	I	S	0
92A	S	S	S	S	S	S	S	S	S	0

R represents Resistance, S represents Sensitive and I represents Intermediate.

**– = antibiotics not tested.**

### 3.2 UHP46 inhibited S46 growth *in vitro*

**Figure 2:**
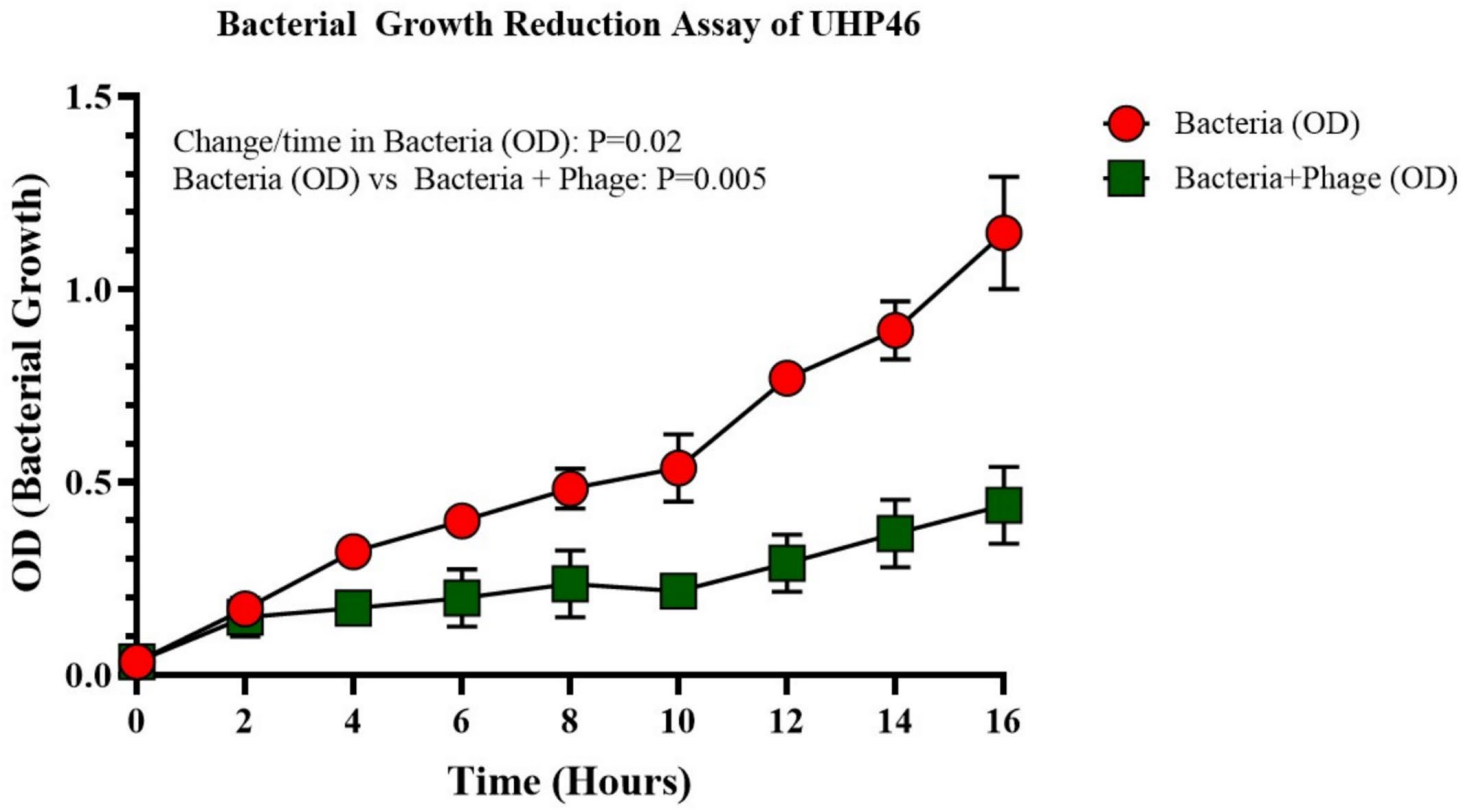
Assay for bacterial growth reduction for UHP46, showing a reduction in *S. aureus* growth in the presence of the UHP46 phage in comparison to the uninfected bacterial cells (control). Results are the mean values from three independent experiments. The reduction of bacteria in the phage treated group is significant having a *p*-value of 0.005.

The phage activity against *S. aureus* was assessed over a 16-h period as shown in Figure 2. Initial OD_600_ values were 0.036 for the control (bacteria only) and 0.034 for the phage-treated sample. Significant differences in bacterial growth emerged over time. After 6 h, OD_600_ was 0.4 for the control and 0.2 for the phage-treated sample. At 16 h, the control reached an OD_600_ of 1.08 while the phage-treated sample was 0.44. These results demonstrate that phages effectively reduced bacterial growth, indicating their therapeutic potential against *S. aureus*.

### 3.3 Thermal stability of phage UHP46

**Figure 3:**
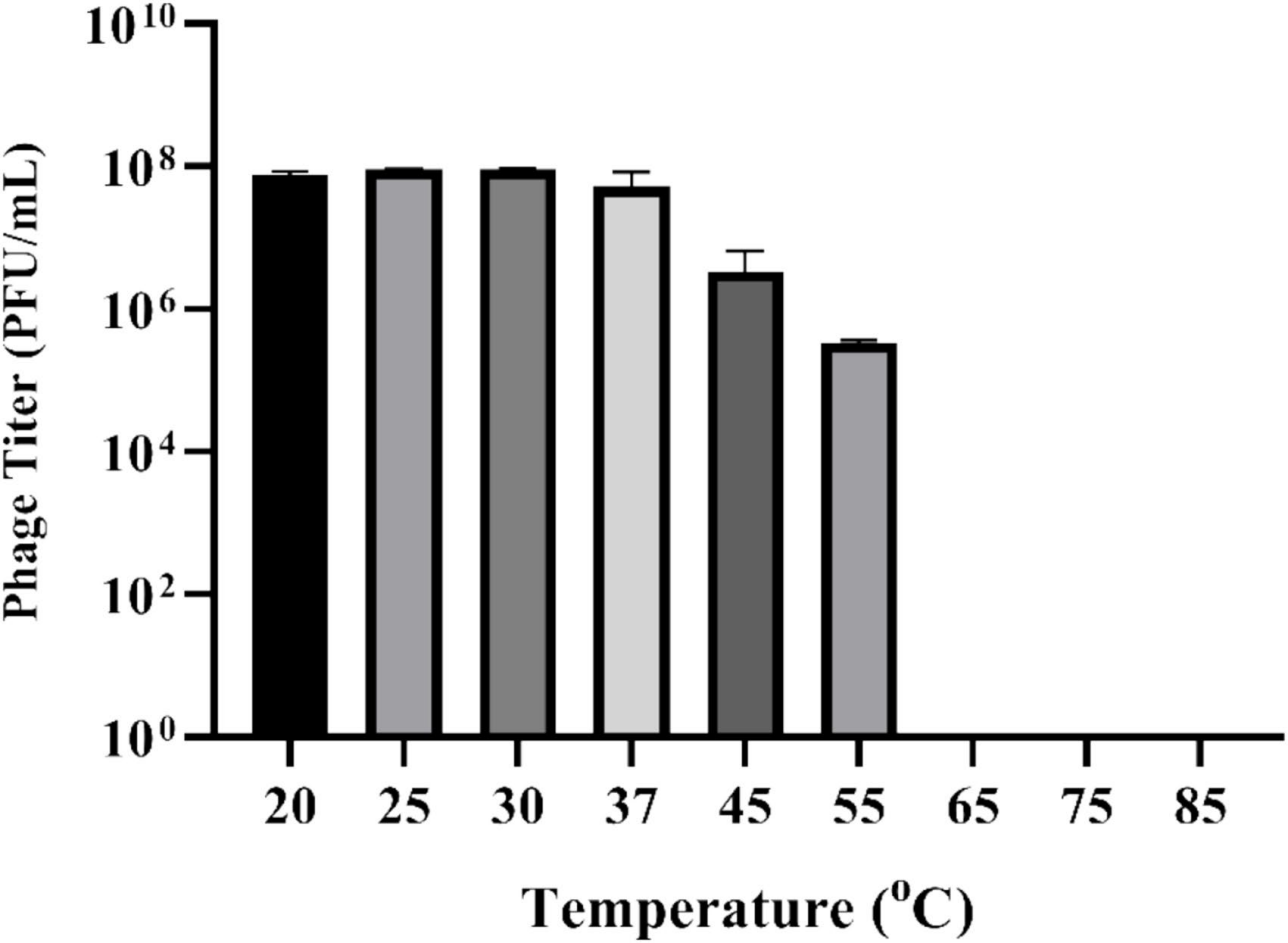
Effect of temperature on stability of phage UHP46. Results are the mean values with SD indicated by error bars from three independent experiments. There is no significant difference in the phage viability from 20 to 37°C.

UHP46 demonstrated stability at 20, 25, 30, 37, and 45°C, with a significant titer decrease at 55°C as seen in Figure 3 (*p*-value < 0.0001). Although UHP46 remained stable at a temperature range of 20–55°C, phages held at 55°C showed the lowest stability, while phages stored at and below 37°C showed the maximum stability.

### 3.4 pH stability of phage UHP46

**Figure 4:**
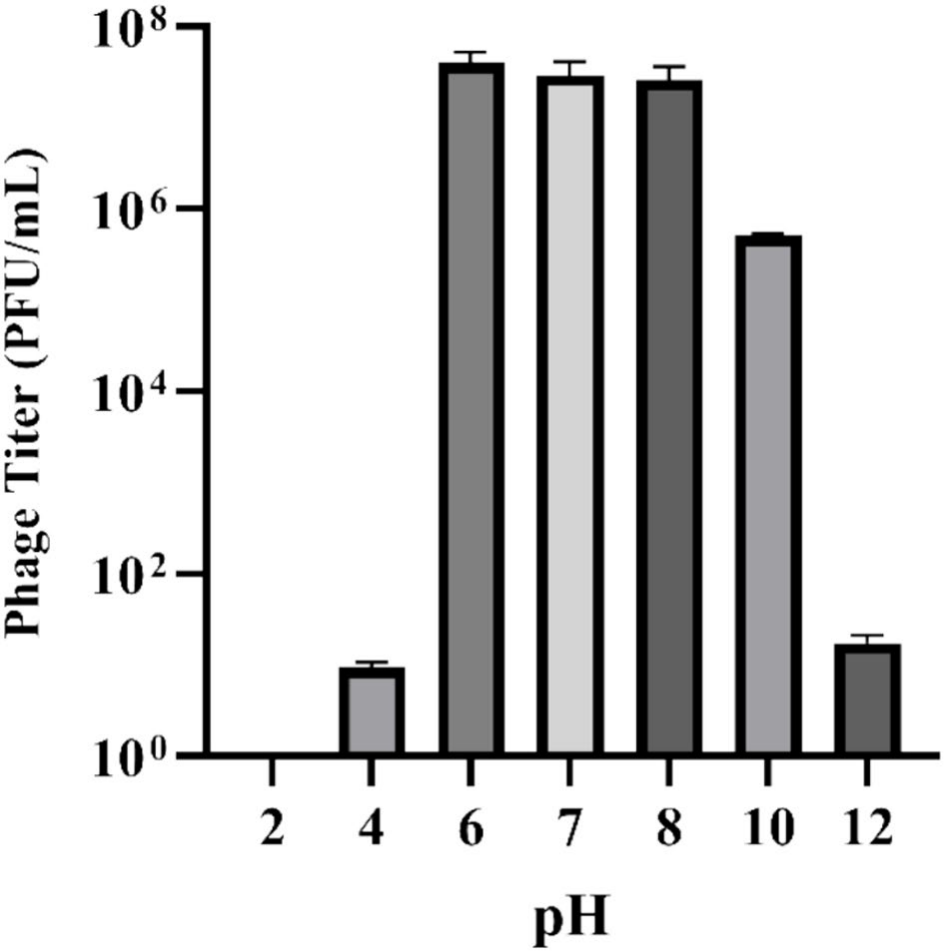
Effect of pH on stability of phage UHP46. Results are the mean values with SD indicated by error bars from three independent experiments. A significant decrease in the phage titer was observed at pH 2 and 12.

Phage UHP46 demonstrated stability at various pH values (4, 6, 7, 8, and 10) as shown in Figure 4. The optimum pH for phage UHP46 was found to be 6–8. At pH 10, a two-log decrease in titer of UHP46 was observed whereas a significant decrease in titer was noticed at pH 4 and 12 (*p*-value < 0.0001). However, no viable phage was found at pH 2.

### 3.5 Latency period and burst size determination

**Figure 5:**
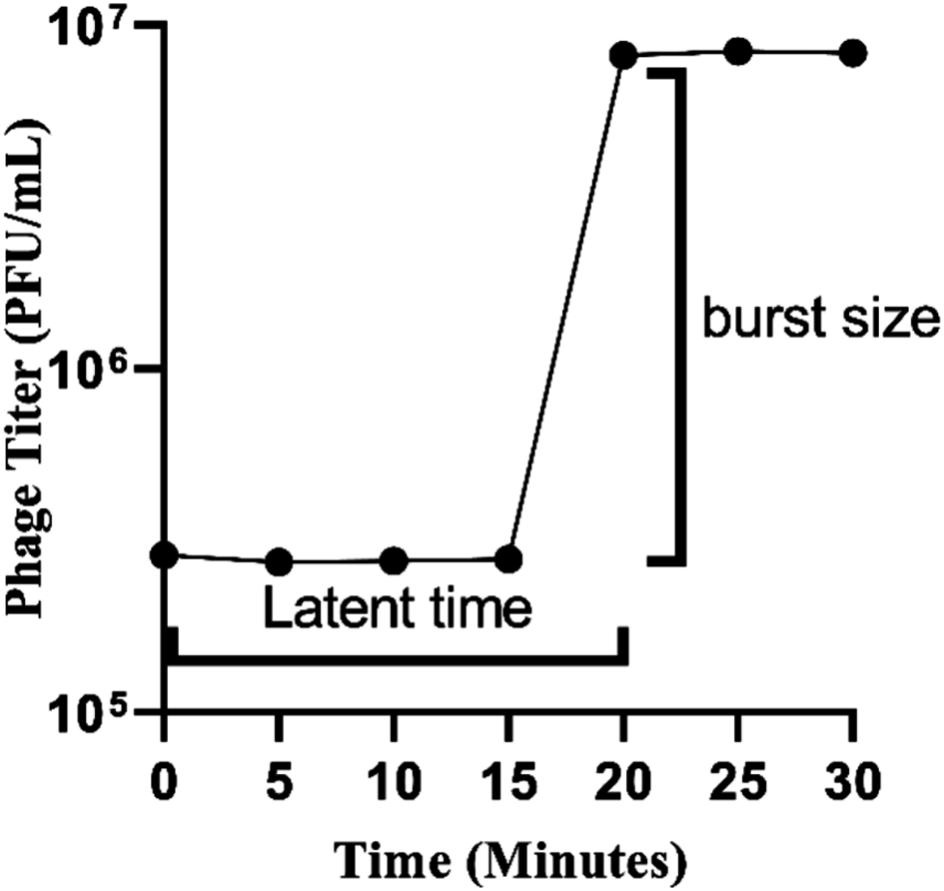
The latent period, or the interval between adsorption and the start of the first burst, was measured in the one-step growth curve assay of phage UHP46. The ratio of the total number of released phage particles to the total number of infected bacterial cells during the latent phase was used to calculate the burst size.

Following the infection of the host cell, a one-step growth curve was established to assess the proliferative ability of phage UHP46. As illustrated in Figure 5, phage UHP46 exhibited a latent period of 20 min and a burst size of 27 ± 1 virions per cell.

### 3.6 Organic solvent stability of UHP46

**Figure 6:**
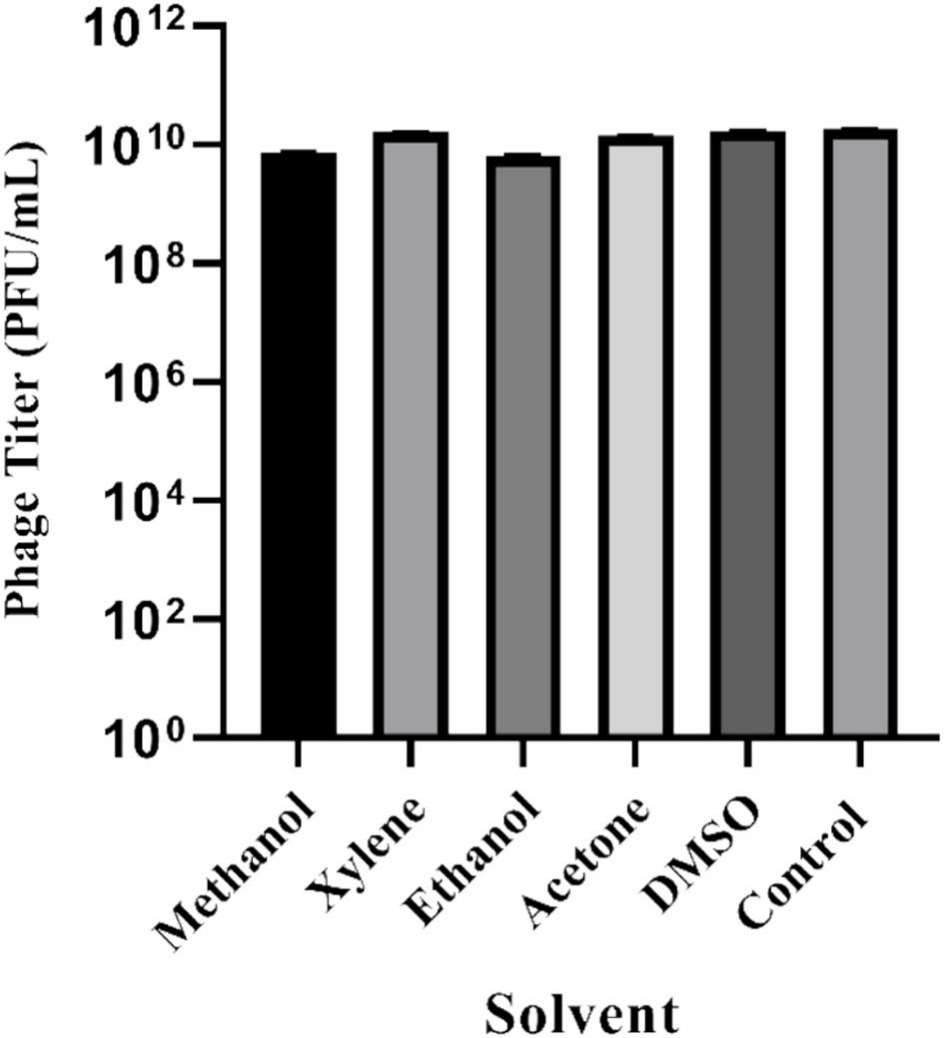
Stability of *S. aureus* phage UHP46 at different polar and non-polar organic solvents. Results are the mean values with SD indicated by error bars from three independent experiments.

UHP46 organic solvent stability under similar exposure conditions to xylene, methanol, ethanol, acetone, and DMSO was observed. Control group exhibited a baseline viability of 1.78 × 10^10^ PFU/mL, with methanol resulting in 7.5 × 10^9^ PFU/mL, xylene showing 1.63 × 10^10^ PFU/mL, ethanol displaying 6.4 × 10^9^ PFU/mL, acetone yielding 1.4 × 10^10^ PFU/mL, and DMSO resulting in 1.73 × 10^10^ PFU/mL (Figure 6). These distinct responses highlight the differential susceptibility of UHP46 to the tested solvents, providing valuable insights into their viability under varied exposure conditions.

### 3.7 Host range of UHP46 phage

Host range analysis of UHP46 demonstrated its lytic activity against 13.7% (4/29) of tested *S. aureus* strains. The analysis revealed clear zones in four of the tested strains, indicating susceptibility to the phage. The detailed results are shown in Table 2. Phage UHP46 was found to be highly selective and specific to a limited number of *S. aureus* isolates.

**Table 2:** Host range of phage UHP46.

*S. aureus*	Spot assay
11	×
16B	×
20A	×
20B	×
25	×
27	×
28	×
30	×
39	×
43	×
44	×
46A	×
S46	✓
46C	×
47B	×
47C	×
48A	×
48B	×
48C	×
49	×
50A	×
50C	×
54B	✓
60B	✓
61B	×
94	✓
90	×
96B	×
92A	×

✓ = clear inhibition zone, × = no inhibition zone.

### 3.8 UHP46 phage stability in milk

**Figure 7:**
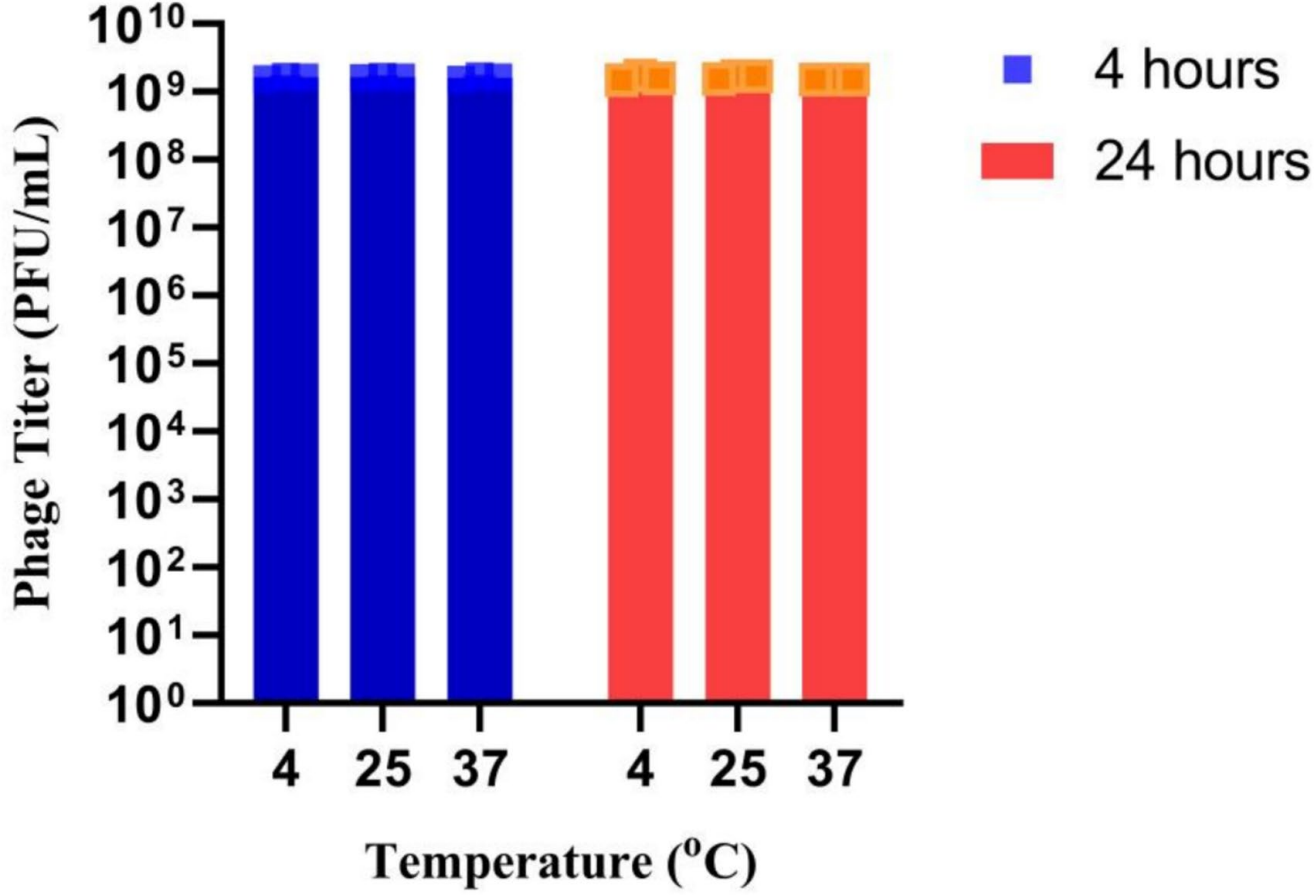
Stability of UHP46 phage in milk. Results are the mean values with SD indicated by error bars from three independent experiments.

UHP46 was found stable at 4°C, 25°C, and 37°C with no significant reduction in phage titer up to 24 h as shown in Figure 7.

### 3.9 Genomic characteristics of phage UHP46

**Figure 8:**
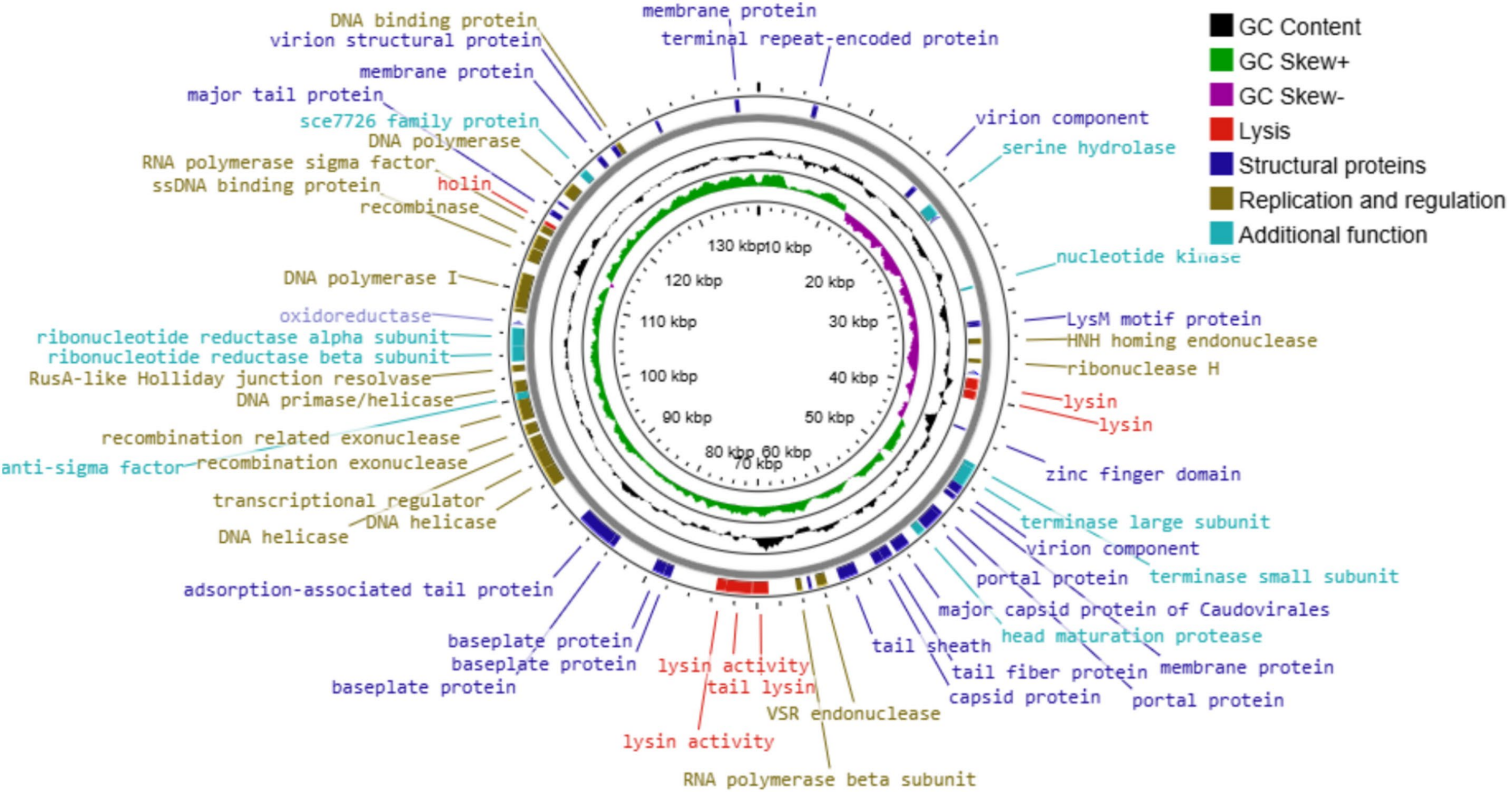
The genome map of UHP46. Circles represent (from the outside) the ORFs, G + C content, GC skew, and genome map scaled in kbp. Putative functional categories are displayed in different colors; lysis (red), structural proteins (dark blue), replication and regulation (brown), and additional function proteins (light blue).

**Figure 9:**
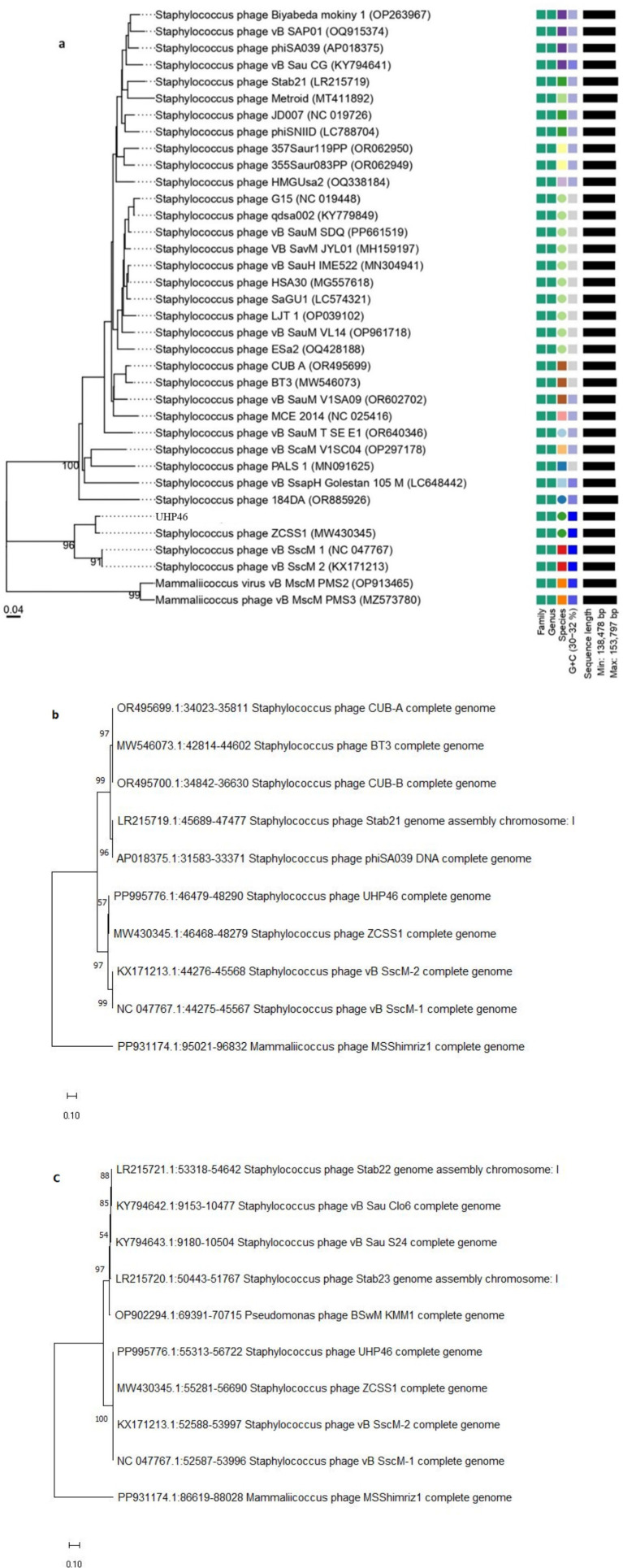
Phylogenetic analysis of UHP46 based on **(A)** complete genome compared with related phages, and nucleotide sequences of **(B)** large terminase subunit, and **(C)** major capsid protein showing relatedness with other phages. The maps were created by using VICTOR database and MEGA11.

UHP46 genome is a double stranded DNA molecule, which is 139,731 bp long and contains G + C content of 31.8%. It has 208 ORFs, among which 72 genes correspond to proteins with identified functions, while 136 ORFs were found uncharacterized, designated as hypothetical. Proteins with predicted functions were included in various categories such as nucleotide metabolism (DNA primase/helicase, RNA polymerase subunit, exonuclease and endonuclease, DNA polymerase), packaging and structural (major capsid protein, tail lysin, tail sheath protein, portal, tail lysin, tail tube, and tape measure and major tail proteins, adsorption-associated tail protein, baseplate protein), and lytic proteins (holin, lysin) (Figure 8). Lytic machinery of UHP46 consists of two phage lysins (encoded by ORF 81 and 82) and one holin encoded by ORF 161. ORF 126 and 31 encode the largest and smallest proteins (hypothetical), with estimated size of ~147 kDa, and ~ 3.8 kDa, respectively. Also, no genes encoding for tRNAs were detected in UHP46 genome. Moreover, 23 genes were found to contain transmembrane helices, while signal P cleavage sites were detected in two genes only (ORF 42 and 60). UHP46 is predicted to be type T5 phage having redundant ends, containing a direct terminal repeat (DTR) region of 12,857 bp. Genome-wide BLAST analysis showed that the UHP46 genome is highly similar to *Staphylococcus* phage ZCSS1 (MW430345), with 97.61% identity and 99% genome coverage. Phylogenetic analysis also revealed the close association of UHP46 with other *Staphylococcus* phages such as, vB SscM 1 (NC 047767), and vB SscM 2 (KX171213) (Figure 9A). Furthermore, there were no genes found coding for integrase, or antimicrobial resistance and virulence factors. Phage AI demonstrated that UHP46 belongs to order *Caudovirales*, family *Herelleviridae*, and genus *Sciuriunavirus*. The lytic nature of phage was further confirmed by the higher virulence scores of 0.85, and 98.83 generated by BACPHLIP, and phage AI, respectively. Phylogenetic analysis performed on the basis of amino acid sequences of large terminase and major capsid protein also showed close association of UHP46 with other *Staphylococcus* phages such as ZCSS1 (MW430345) (Figures 9B,C).

## 4 Discussion

In this work, lytic phage UHP46 was isolated from sewage samples and characterized biochemically. In addition, genomic analysis of UHP46 was performed to determine its suitability as an anti-*Staphylococcal* against bovine mastitis isolates. Over a 16-h test period, UHP46 effectively reduced the growth of *S. aureus*. The growth of the bacteria in the presence of phage after 16 h was equal to 3 h growth without phage (Figure 2). This explains the significance of the phage UHP46 to control the *S. aureus*. Previously, phage SPW has demonstrated strong bacteriolytic activity against *S. aureus* by inhibiting its growth up to 60 h (Kala et al., 2014). One key feature of potential therapeutic phages is to target highly specific pathogenic bacteria, however, high specificity leads to comparatively narrow host range (Zhang et al., 2015). Phage UHP46 showed a narrow host range, infecting only 13.7% of the tested *S. aureus* strains. However, the host range of phage can be further improved through receptor modifications, or by using cocktail of phages as reported previously (Abedon et al., 2021; Dunne et al., 2021).

The physio-chemical factors such as temperature, and pH affect phages’ infectivity, storage viability, and incidence which is crucial for the success of phage therapy (Pradeep et al., 2022). Phage UHP46 showed good viability at various temperatures (20–55°C), while viability at and below 37°C was found to be maximum (Figure 3). Kraushaar et al. (2013) also reported good stability of three *S. aureus* phages (PSa1, PSa2, and PSa3) up to 50°C after 1-h treatment at thermal stress (Kraushaar et al., 2013). Moreover, UHP46 demonstrated stability at various pH values (4–10), with maximum phage activity observed at neutral pH (Figure 4). These results are similar to another study which reported stability of *S. aureus* phage vB_SauS_IMEP5 at pH 3–12 and inactivity of phage at pH 2 (Zhang et al., 2017). Moreover, WV phage showed highest viability at 6–7 pH range, and decreased stability at pH 3–4 (Jiang et al., 2021). Hence, tolerance of phage UHP46 to different environmental conditions indicates its potential to work effectively at normal or near normal body temperature and pH. Furthermore, different organic solvents are used in phage formulations, which necessitates the understanding of any potential harmful effects of these reagents on the stability of phages (Cao et al., 2023). Phage UHP46 retained activity in xylene, acetone, and DMSO, while stability was declined significantly in alcohols including methanol and ethanol. In a similar study, Oduor et al. (2020) reported the inactivation of *S. aureus* phages Stab20, Stab21, Stab22, and Stab23 in ethanol solution with concentration above 25% vol/vol (Oduor et al., 2020). The viability of *E. coli* phages was also reported to decrease after exposure to alcohols (Yamashita et al., 2000; Padmesh et al., 2023). It has been suggested that water-miscible organic solvents like alcohol may disrupt the hydrophobicity-hydrophilicity balance of the phage surface proteins, leading to disruption of phage structure and reduced viable phage titer (Cao et al., 2023). Phage stability in DMSO has been shown to vary significantly. The effect of DMSO treatment on two *P. aeruginosa* phages was investigated, with PPaMa3/19 maintaining 87.7% stability, while PPaMa4/19 lost infectivity with only 4.8% stability (Majdani and Hatefirad, 2022). Phage UHP46, however, remained viable over DMSO treatment, indicating its potential to sustain in environments where DMSO is used as a solvent or preservative. Phage UHP46 also showed good stability in acetone as reported in previous similar studies (Pallavi et al., 2021). Moreover, UHP46 was found stable after treatment with xylene, indicating its potential to be preserved in diverse range of solvents. The stability profile of UHP46 in above solvents may help in the selection of appropriate solvents for the optimization of phage formulation in therapeutic applications. Furthermore, UHP46 showed good stability in milk over a 24-h test period which indicates its potential to be used in milk applications. Similarly, significant stability of *Salmonella* phage ZCSE6 was reported when the milk was stored at 4°C for 6 days (Abdelsattar et al., 2021). Burst size and the latent period is an important criterion for phage to be used as therapeutic. Phages with the low latent period are considered good. UHP46 proliferates effectively in the targeted *S. aureus* strain, exhibiting latent period of 20 min, and a burst size of 27 ± 1 progeny/cell. In another study, *S. aureus* phage JD419 demonstrated burst size of 33 phages generated per cell (Feng et al., 2021).

Based on the genomic analysis, UHP46 is a member of the *Herelleviridae* family. *Herelleviridae* (formerly a part of *Myoviridae*) phages infecting *S. aureus* strains are commonly used for therapeutic purposes due to their effective lysis ability against MDR pathogens (Kornienko et al., 2023). The genome of UHP46 contains 139,731 bp, which is within the range of other *Herelleviridae* phages characterized against *S. aureus*, such as the 137,950 and 138,307 bp genomes of KSAP7 and KSAP11, respectively (Kitamura et al., 2020). Whole genome comparison revealed that UHP46 has 95–97% nucleotide sequence identity to *Staphylococcus* phages vB_SscM-1 (NC_047767.1), vB_SscM-2 (KX171213.1), and ZCSS1 (MW430345.1). The UHP46 genome carries terminal redundancies in the form of 12,857 bp direct terminal repeats, indicating that UHP46 follows T5 phage packaging model (long DTR) as reported in case of *Staphylococcus* phage vB_SauM_VL10 (Lerdsittikul et al., 2024). In this packaging system, terminase recognizes the specific sequences on the phage DNA where DTR are generated (Garneau et al., 2017), and it also minimizes the possibility of the unwanted host transduction of virulence or AMR genes (Pradal et al., 2023). In fact, UHP46 genome showed no virulence, toxin or antibiotic resistance genes, suggesting its potential to be used safely in clinical trials.

The majority of the UHP46 encoded proteins are hypothetical. This is commonly observed in annotated phage genomes, and the possible reasons may include the low characterization of phage proteins, and the wide phage diversity (Pradal et al., 2023). The UHP46 genes with predicted biological functions could be categorized into different groups such as replication and metabolism-associated genes, lysis genes, and structural genes. Multiple genes involved in replication and metabolism were identified in the UHP46 genome including RNA polymerase, and DNA polymerase which suggest that UHP46 is independent of the host machinery for transcription and DNA replication. Additionally, ORF142 was predicted to be a bifunctional protein DNA helicase/primase which is responsible for the unwinding and catalytic synthesis of DNA (Nazir et al., 2022). ORF72 encodes HNH endonuclease, which have been reported to play crucial role in infection, reproduction, and life cycle of phages (Kala et al., 2014). UHP46 genome also contains genes encoding RusA-like Holliday junction resolvase (ORF145) and VSR endonuclease (ORF111), which cleave the phosphodiester bonds between the DNA nucleotides, and these proteins are presumed to provide nucleotide monomers required for phage DNA synthesis (Yang et al., 2021).

Among the ORFs, different UHP46 genes were predicted to be involved in the host cell lysis. ORF81 and 82 encode for phage lysin, N-acetylmuramoyl-L-alanine amidase, which disrupts the bacterial peptidoglycan. The UHP46 genome also contains a holin gene, two tail proteins with lysin activity, and a tail lysin. Double-stranded DNA tailed phages (*Caudovirales*) employ a cassette consisting of holin and endolysin to kill bacterial cells (Wu et al., 2021). Holins enable endolysins to reach the peptidoglycan layer of bacterial cell wall by creating pores in the cell membrane of bacteria. As a result, new phage progeny is released to further infect the other bacterial cells (Lu et al., 2020). Moreover, the G + C content of UHP46 (31.8%) phage was also comparable to that of other *S. aureus* phages such as vB_SauS_SA2 (31.9%) (Wang et al., 2019). Additionally, two genes encoding terminase large and small subunit proteins were found in the UHP46 genome, and both subunits are considered to form a complete terminase (Liao et al., 2008). Terminase large subunit, a DNA packaging protein, is used for phylogenetic classification of phages due to its universally conserved sequence (Mardiana et al., 2022). Phylogenetic analysis of UHP46 large terminase and major capsid protein indicated its close association with the Staphylococcus phage ZCSS1 (MW430345), which is also classified in *Herelleviridae* family.

## 5 Conclusion

This study investigated the possibility of using the virulent phage UHP46 as a therapeutic agent against *S. aureus* strains sourced from bovine milk. Morphological and genomic analyses indicated that phage UHP46 resembles members of the *Herelleviridae* family and lacks any toxin or virulence genes. Based on these findings, we conclude that phage UHP46 could be an effective phage application for targeting *S. aureus* in bovine mastitis.

## Data Availability

The datasets presented in this study can be found in online repositories. The names of the repository/repositories and accession number(s) can be found at: https://www.ncbi.nlm.nih.gov/genbank/, PP995776.

## Footnotes

^1^ https://www.ebi.ac.uk/interpro/search/sequence/

^2^ https://blast.ncbi.nlm.nih.gov/Blast.cgi?PROGRAM=blastp&PAGE_TYPE=BlastSearch&LINK_LOC=blasthome

^3^ https://dtu.biolib.com/DeepTMHMM

^4^ https://services.healthtech.dtu.dk/services/SignalP-5.0/

^5^ https://lowelab.ucsc.edu/cgi-bin/tRNAscan-SE2.cgi

^6^ https://web.expasy.org/protparam/

^7^ https://cpt.tamu.edu/galaxy-pub

^8^ https://ggdc.dsmz.de/victor.php#

^9^ https://www.megasoftware.net/dload_win_beta

^10^ https://app.phage.ai/

^11^ https://phagenomics.net/phagenomics_portal/

^12^ http://genepi.food.dtu.dk/resfinder

^13^ https://cge.food.dtu.dk/services/VirulenceFinder/
